# Compositions and Anti-Tumor Activity of *Pyropolyporus fomentarius* Petroleum Ether Fraction In Vitro and In Vivo

**DOI:** 10.1371/journal.pone.0109599

**Published:** 2014-10-10

**Authors:** Yanhua Zhang, Yaping Xiao, Pan Wang, Quanhong Liu

**Affiliations:** 1 Key Laboratory of Medicinal Resources and Natural Pharmaceutical Chemistry, Ministry of Education, National Engineering Laboratory for Resource Developing of Endangered Chinese Crude Drugs in Northwest of China, College of Life Sciences, Shaanxi Normal University, Xi'an, Shaanxi, China; 2 College of Life Sciences; Jiangsu Normal University, Xuzhou, Jiangsu, China; National Cheng Kung University, Taiwan

## Abstract

The chemical compositions and anti-tumor activities of the petroleum ether fraction (PE), from mushroom *Pyropolyporus fomentarius*, were studied. Upon gas chromatography–mass spectrometry (GC–MS) analysis, nine major constituents were identified in the fraction. In vitro, the PE showed cytotoxic activity against murine sarcoma S180 (S180) cells in a dose- and time-dependent manner, and the cytotoxic effects were associated with apoptosis. The mitochondrial membrane potential loss and the intracellular ROS generation were greatly increased in the *Pyropolyporus fomentarius* PE treated group, suggesting cell apoptosis, induced by the PE in S180 cells, might be mitochondria dependent and ROS mediated. Consistent with in vitro findings, the in vivo study showed that the *Pyropolyporus fomentarius* PE was also effective in inhibiting the tumor growth induced by S180 cells and had lower immune organ toxicity. We found that the *Pyropolyporus fomentarius* PE has significant anti-tumor activity and great potential in screening anti-tumor drugs.

## Introduction

Cancer has become the leading cause of death worldwide [1]–[3]. Compared with 12.7 million and 7.6 million in 2008, respectively, an estimated 14.1 million new cancer cases and 8.2 million cancer-related deaths occurred in 2012. Projections based on the GLOBOCAN 2012 estimates projected that new cancer cases will rise to 19.3 million per year by 2025, due to the growth and ageing of the global population [4]. Chemotherapy is still the main clinical treatment for cancer. Most of the chemotherapeutic drugs induce serious multi-drug resistance and a series of side effects, e.g., fatigue, muscle and joint pain, impaired immune responses, anemia, neutropenia and thrombocytopenia [5]–[8]. Therefore, searching for novel anti-tumor agents from natural products with fewer adverse effects is highly important.

The use of medicinal mushrooms in the fight against cancer has been known for a very long time in Korea, China, Japan, Russia, USA and Canada. Mushrooms produce a variety of complex, low-molecular-weight compounds with diverse chemical compositions, such as phenolic compounds, polyketides, triterpenoids and steroids [9], [10]. Many have shown direct beneficial effects on cancer development by interfering with specific transduction pathways [11], [12].

Mushroom *Pyropolyporus fomentarius* (L. ex Fr.) Teng (*P*. *fomentarius*), also called *Fomes fomentarius*, is a fungus of the Polyporaceae family that acts a parasite on beech and birch trees [13]; it has worldwide distribution [14]. *P*. *fomentarius* has wide-ranging uses, including medicinal use [15]–[19]. Many bioactive substances were isolated from the *P*. *fomentarius* petroleum ether fraction, such as β-sitosterol, 5α,8α-epidioxy-ergosta-6,22-dien-3β-ol, ergosta-7,22-dien-3β-ol [20], ergosta-7,22-dien-3-palmitate, Stearic acid, Palmitic acid, Ergosta-7,22-dien-3-one, ergosta-7,22-dien-3-one, dimethyl acetal [21] and sterols [22]. However, there no studies have demonstrated its anti-tumor activities and the underlying mechanism.

Many traditional herbal medicines have shown antiproliferative effects on the S180 cell line [23], [24] or cytotoxic activities on the S180-bearing mouse model, alone [24]–[29] or combined with cyclophosphamide (CTX) [30]. So, in the present study, we use S180 cell line to investigate the anti-tumor efficacy of *P*. *fomentarius*. The purpose of this study was to identify the bioactive compounds from the petroleum ether fraction using gas chromatography–mass spectrometry (GC-MS) analysis as well as to evaluate the potential anti-tumor efficacy and the mechanism of the fraction by testing the (1) cell cytotoxicity, (2) apoptosis rates, (3) morphological changes, (4) reactive oxygen species (ROS) generation and mitochondrial membrane potential (MMP) fluctuation in murine sarcoma S180 (S180) cells and (5) the tumor inhibitory effect on S180-bearing mice.

## Materials and Methods

### Plant materials

Sampling in the Qinling Mountain area of Shaanxi Province was permitted by the management committee of Qinling Nature Protection Area. The field studies did not involve endangered or protected species.

We collected the sporophores of *P. fomentarius* from Pinghe-liang, south of QinLing Mountains, Shaanxi province, China (latitude, 33°27′ N; longitude, 108°30′ E; altitude, 2305 m). The sporophores were identified by Prof. Yaping Xiao in the Ministry of Education, Key Laboratory for Medicinal Plant Resource and Natural Pharmaceutical Chemistry, Shaanxi Normal University, Xi'an, Shaanxi, P.R. China.

### Preparation of the petroleum ether fraction

At 45°C, crushed plant material was homogenized in 95% ethanol (EtOH, w/v), using ultrasonic-assisted extraction (UAE), three times (each for 30 min). The filtrate was vacuum concentrated and freeze-dried after filtration through filter paper. The extract was then fractionated with petroleum ether. The petroleum ether fraction was obtained by evaporation under reduced pressure. Then, the fraction was homogenized in 95% ethanol to obtain a stock solution of 120 mg/ml, which was passed through a 0.22 µm filter for use in subsequent cell experiments. For the animal experiment, the petroleum ether fraction was presolubilized in ethanol and prepared as a fine suspension in 1% gum acacia; the final concentration of the ethanol was maintained at 1% (v/v). Note that the *P. fomentarius* petroleum ether fraction was called PFPE in this study.

### Cell culture

S180 cells were obtained from the Cell Bank of the Chinese Academy of Science, Shanghai, China. Supplemented with 10% fetal bovine serum (FBS, Hyclone, USA), 1% penicillin–streptomycin (100 U/ml penicillin and100 µg/ml streptomycin) and 1% glutamine, the cell line was cultured in RPMI-1640 (Gibco, Life Technologies, Inc., USA). Cells were maintained at 37°C with a humidified 5% CO_2_ atmosphere.

### Assay for cytotoxicity

The cytotoxic activity of PFPE on S180 cells was measured with the 3-(4,5-Dimethylthiazol-2-yl)-2,5-diphenyltetrazolium bromide (MTT) assay. Healthy human epithelial kidney cells (HEK-293) were used as normal cells [31], [32] by contrast to examine the cytotoxic effect of the PFPE. Briefly, the control or PFPE-loading cells at the density of 5×10^4^ cells/ml (100 µl) were seeded in 96 well culture plates, and cell viability after different incubations (24 and 48 h) was determined by adding 10 µl of MTT solution (5 mg/ml in PBS) and the mixture was incubated for an additional 4 h at 37°C in a CO_2_ incubator. The formazan crystals were dissolved in 100 µl of 10% SDS, 5% isobutyl alcohol, and 0.01 mol/L HCL solution, and the absorbance at 570 nm was recorded using a micro-plate reader (BIO-TEK ELX800, USA). The cell survival rate was obtained by comparison with the control.

### Annexin V-FITC/PI staining experiment

Apoptotic cells were measured with an Annexin V-FITC Detection Kit (Invitrogen, USA) according to the manufacturer's protocol. Briefly, cells at 5×10^4^ cells/ml were treated with various concentrations (0, 120, 240 and 480 µg/ml) of PFPE for 36 h at 37°C. Cells were then harvested and re-suspended in the binding buffer. Cells were stained with 10 µl of Annexin V-FITC and 5 µl of propidium iodide (PI) for 15 min at room temperature in the dark. The apoptotic index was immediately determined by flow cytometry.

### DNA fragmentation assay

Krysko et al. describe a way to analyze DNA fragmentation by adding PI to the dying cells based on flow fluorocytometric detection [33]. By freeze-thawing, PI intercalates in the DNA and the size of DNA fragments appears as a hypoploid DNA histogram. To detect the effect of the PFPE on the DAN damage of S180 cells, we carried out oligonucleosomal DNA fragmentation by flow fluorocytometry. Cells at 5×10^4^ cells/ml were treated with various dose of PFPE for various time periods. Then, cells were collected and stained with 5 µg/ml PI (Sigma, St. Louis, USA) and analyzed the DNA content with flow cytometry (Guava easyCyte 8HT, Millipore, USA).

### 4',6-diamidino-2-phenylindole (DAPI) staining

DAPI staining was performed to detect changes of nuclei morphology of tumor cells. S180 cells were seeded at 5×10^4^ cells/ml and treated with different concentrations (0, 120, 240 and 480 µg/ml) of the PFPE for 24 h at 37°C. Then, cells were collected and stained with 4 µg/ml of DAPI (Sigma, St. Louis, USA) for 30 min at room temperature. After cleaning with PBS, samples were stored at 4°C in the dark and viewed under a fluorescence microscope (Nikon, Japan).

### Mitochondrial membrane potential

The MMP was measured by the uptake of the mitochondrial specific lipophilic caption dye rhodamine 123 (Rh123). After various incubation times post the PFPE-loading, S180 cells were collected by centrifugation for 5 min and washed with PBS; then incubated with 2 µg/ml Rh123 for 30 min at room temperature, washed and re-suspended in PBS buffer. Cells were immediately analyzed by flow cytometry (Guava easyCyte 8HT, Millipore, USA). The excitation wavelength was 488 nm and emission wavelength was 530 nm. Histograms were analyzed using FCS Express V3.

### Detection of the intracellular reactive oxygen species generation

In order to detect changes of the intracellular ROS level, we measure the oxidative conversion of fluorescent probe 2′,7′-dichlorofluorescein-diacetate (DCFH-DA) (Invitrogen, CA, USA) to fluorescent 2′,7′-dichlorofluorescein (DCF). Within the cell, DCFH-DA is enzymatically hydrolyzed to form nonfluorescent DCFH, in the presence of ROS, which is then rapidly oxidized to form fluorescent DCF, and the fluorescence intensity of DCF is proportional to ROS production. Cells (5×10^4^ cells/ml) incubated with various concentrations of the PFPE for various time periods, were harvested and stained with 10 µM DCFH-DA, and were then incubated at 37°C for 30 min in the dark. After washing with PBS, cells were immediately analyzed by flow cytometry (Guava easyCyte 8HT, Millipore, USA). The excitation wavelength was 488 nm and emission wavelength was 530 nm. Histograms were analyzed using FCS Express V3.

### Animals and treatment

The Institute of Cancer Research (ICR) mice (female, 18–20 g body weight) were supplied by the Experimental Animal Center of Xi'an Jiao Tong University (Xi'an, China). Mice were maintained at room temperature (18–24°C), with a relative humidity of 70±10% and a 12 h light-dark cycle. In accordance with the guidelines established by the National Science Council of Republic China, mice were housed and cared for. After being fed in our facility for 1 week, all mice were inoculated with 0.1 ml of S180 sarcoma cells (1×10^7^cells/ml) into the left oxter region of ICR mice and randomly divided into 4 groups of 6 animals in each group. After 24 h of tumor inoculation, the PFPE fraction was administered orally at doses of 120 and 240 mg/kg body weight once per day, respectively. The group, administered with vehicle alone (ethanol-gum acacia, p.o.), was maintained as the model control. Cyclophosphamide (20 mg/kg body weight, i.p.) was used as the standard reference drug.

There was no rejection response after tumor cell inoculation in ICR mice. At the end of the third week after tumor induction, animals were sacrificed by cervical dislocation under anesthesia using excessive diethyl ether, and tumor development was evaluated by determining the tumor mass (g) and calculating the inhibition rate [34]. The inhibition rate was calculated as: (1-average tumor weight of treated group/average tumor weight of the control group) ×100%. Meanwhile, the thymus gland and spleen were excised and weighed (mg) to calculate the thymus and spleen indexes. The thymus and spleen indexes were calculated as thymus weight/bodyweight ×100% and spleen weight/bodyweight ×100%, respectively.

This study was approved by the Institutional Animal Care and Use Committee (IACUC) of Chinese Academy of Sciences. All experiments with live animals were carried out with the approval of the Committee on the Ethics of Animal Experiments of the Shaanxi Normal University. And, all all efforts were made to minimize suffering.

### Component analysis by GC/MS

To investigate the composition of the PFPE, GC–MS analysis was performed, with a 7890A GC/5975C MS system (Agilent, USA) fitted with a fused silica capillary column (HP-5 MS, 30 m×25 mm ID, 0.25 µm film thickness; Agilent J&W Scientific, Folsom, CA, USA). One microliter of the sample was analyzed, with a split ratio of 10∶1 (v/v). The temperature rise programs had an initial temperature of 50°C for 2 min, which was raised 10°C/min to 200°C and then maintained for 2 min; raised to 250°C at 5°C/min and then maintained for 6 min; and raised to 280°C at 10°C/min and then maintained isothermally for 5 min. The injector temperature was set at 280°C and the interface temperature was 150°C. The MS source Helium was used as the carrier gas at a flow rate of 1 ml/min. The ionization source temperature was 250°C. Mass spectrometry was determined by the full-scan method, ranging from 50 to 600 (m/z). Metabolites were identified by comparison with the NIST Mass Spectral Search Program 2008 database (version 2.0, FairCom Co., Columbia, MO, USA).

## Statistical Analysis

Data were presented as the mean ± standard deviations from at least three independent experiments. Differences among the groups were assessed with one-way analysis of variance, p<0.05 was considered to be significant and p<0.01 was considered to be extremely significant.

## Results

### Effect of the PFPE on cell viability

**Figure 1:**
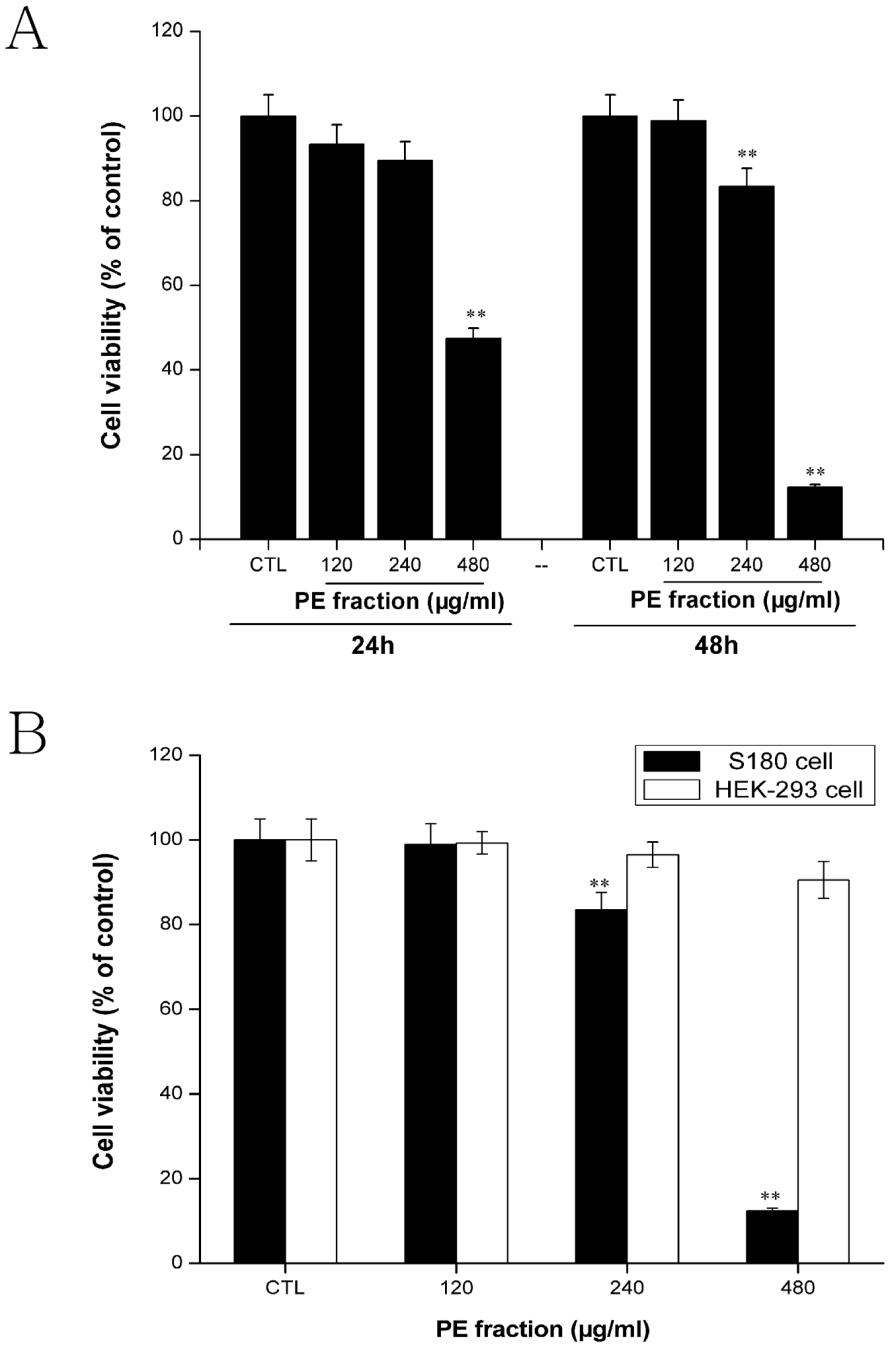
Cytotoxicity effect of *Pyropolyporus fomentarius* PE fraction. (A) S180 cells were treated with 0, 120, 240 and 480 µg/ml of PE fraction for 24 h, 48 h. (B) Cell proliferation of PE fraction between HEK 293 cells and S180 cells at 48 h. Each value is expressed as a mean ±SD of three independent experiments. **p<0.01 versus the control (CTL).

S180 cells at a density of 5×10^4^ cells/ml were incubated with the indicated concentration of PFPE for 24 h and 48 h. The MTT assay revealed that different concentrations of the PFPE induced time-dose-dependent inhibition effects (Figure 1A), with the exception of the 120 µg/ml treatment. The IC50 values were approximately 528.12 and 318.43 µg/ml when cells were incubated with the PFPE fraction for 24 and 48 h, respectively. Interestingly, HEK-293 cell line was less susceptible under the applied concentrations of the PFPE (Figure 1B).

### Apoptosis assessment

**Figure 2:**
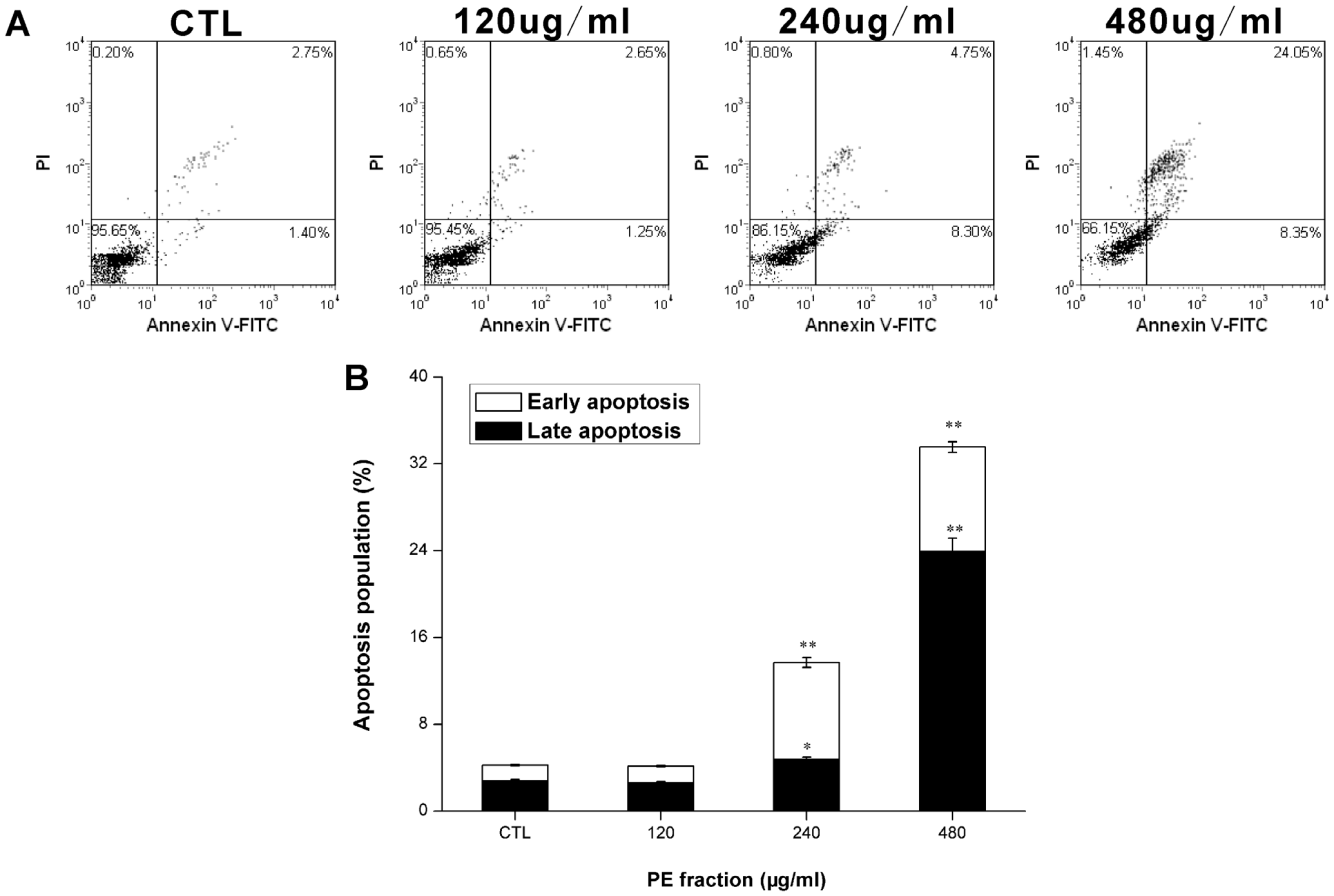
Apoptosis-inducing effect of *Pyropolyporus fomentarius* PE fraction on S180 cells as detected by Annexin V-FITC (AV)/PI method. (A) cells were analyzed at 36 h post-treatment by flow cytometry. Dot-plot graphs show viable cells (AV^−^/PI^−^), early apoptotic cells (AV^+^/PI^−^), late apoptotic cells(AV^+^/PI^+^), and necrotic cells (AV^−^/PI^+^). (B) The ratio of early apoptotic cells and late apoptotic cells are represented as means ±SD (n = 3). *p<0.05 and **p<0.01 versus the control (CTL).

Using Annexin V-FITC/PI double staining, we analyzed phosphatidylserine exposure as an early marker of apoptosis. As shown in Figure 2A, after 36 h treatment in the control group, the viable cells were 95.65%, the cell population in the early stage of apoptosis (lower right) was 1.40% and the cell population in the late stage of apoptosis (upper right) was 2.75%. However, in treatment group, when the drug concentration increased from 240 µg/ml to 480 µg/ml, the apoptotic cell population (early and late stage of apoptotic cells) increased from 13.05 to 32.40%, which showed, compared with control, the percentages of cells with Annexin V-positive staining increased gradually (p<0.05, p<0.01) (Figure 2B), suggesting the PFPE could stimulate an apoptotic response in the S180 cells.

### PFPE induced cell morphological changes

**Figure 3:**
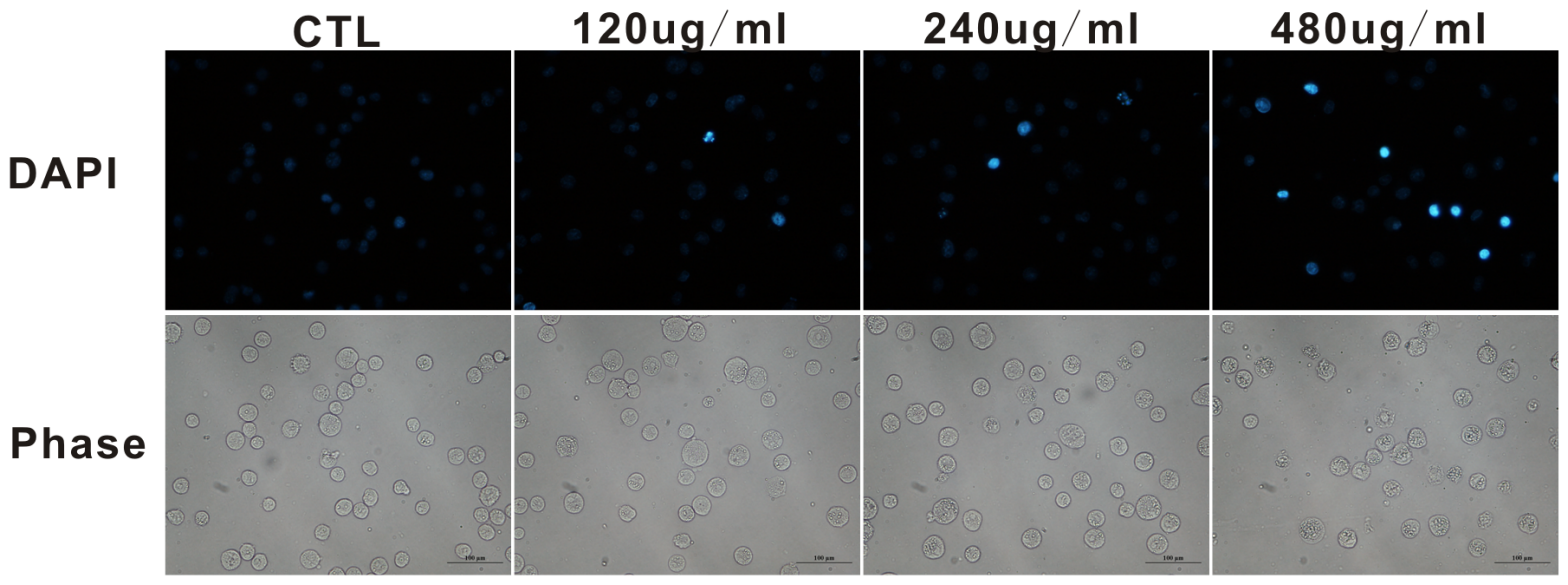
Apoptosis observed by DAPI staining. Cells were treated with PE fraction at the indicated concentrations for 24 h, fluorescent and phase contrast images were captured at the same field. (Bar  = 100 um).

Differences in the cell morphology between the PFPE-treated and control group were examined by DAPI staining. As shown in Figure 3, at 24 h after incubation in control cells, the nuclear DAPI staining was slightly blue and homogeneous, and the contrast phase indicated normal rotundity cell morphology. However, in the treatment group, especially the 480 µg/ml group, cells exhibited morphological features of apoptosis, such as condensed chromatin with bright nuclear DAPI staining, and the phase images also suggested that the cell morphology was seriously damaged.

### DNA fragmentation assay

**Figure 4:**
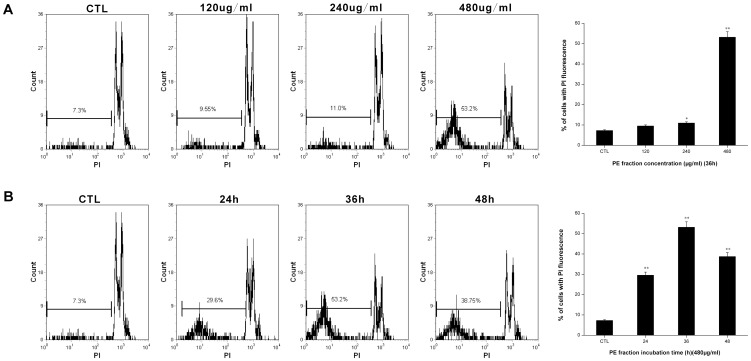
DNA fragmentation assay of S180 cells exposed to *Pyropolyporus fomentarius* PE fraction. Cells were treated with different concentrations of PE fraction for 36 h (A), and treated at 480 µg/ml dose for various time periods (B), and stained with PI and analyzed by flow cytometry. Histograms show number of cell channel (vertical axis) vs. PI fluorescence (horizontal axis). Each value is expressed as a mean ±SD of three independent experiments. *p<0.05 and **p<0.01 versus the control (CTL).

To confirm DNA damage induced by PFPE in S180 cells, the DNA fragmentation assay was performed by flow cytometry as described in the methods. As indicated in Figure 4A, cells, in the treated group showed dose-dependent DNA damage. Meanwhile, in the 480 µg/ml PFPE treated group, the percentage of cells with higher PI fluorescence gradually increased after 24 h treatment, which peaked at 36 h, but additional incubation up to 48 h did not produce more DNA fragments than the 36 h incubation (Figure 4B). These results implied that the PFPE markedly induced DNA damage in S180 cells.

### Mitochondrial membrane potential assay

**Figure 5:**
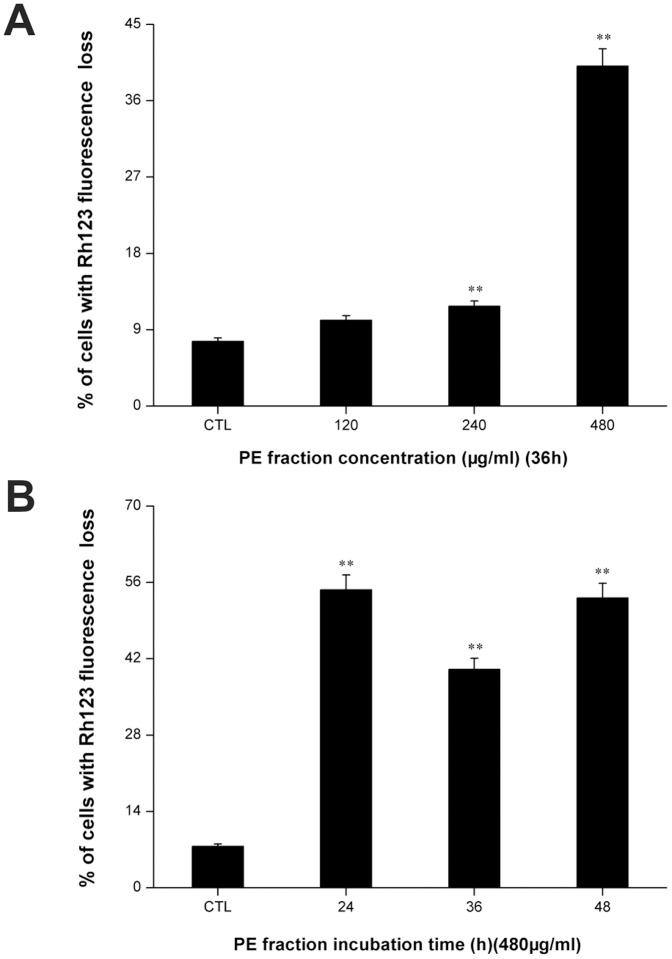
Effect of *Pyropolyporus fomentarius* PE fraction on the mitochondrial membrane potential of S180 cells. Cells were treated with different concentrations of PE fraction for 36 h (A), and treated at 480 µg/ml dose for various time periods (B), and stained with PI and labeled with rhodamine 123, analyzed by flow cytometry. Each value is expressed as a mean ±SD of three independent experiments.**p<0.01 versus the control (CTL).

The depletion of MMP is an early marker of the apoptotic process. To determine whether an early loss of MMP occurred during treatment with the PFPE in S180 cells, we performed MMP measurement using Rh123 staining. As shown in Figure 5A, the percentage of cells with Rh123 fluorescence loss significantly increased in a dose-dependent manner compared with control (p<0.01). The Rh123 fluorescence loss in treated cells was an early event. At 24 h after incubation, the percentage of cells with Rh123 fluorescence loss was 54.65% (Figure 5B). These findings provide strong evidence that PFPE caused the disruption of MMP in S180 cells.

### PFPE induces intracellular ROS generation

**Figure 6:**
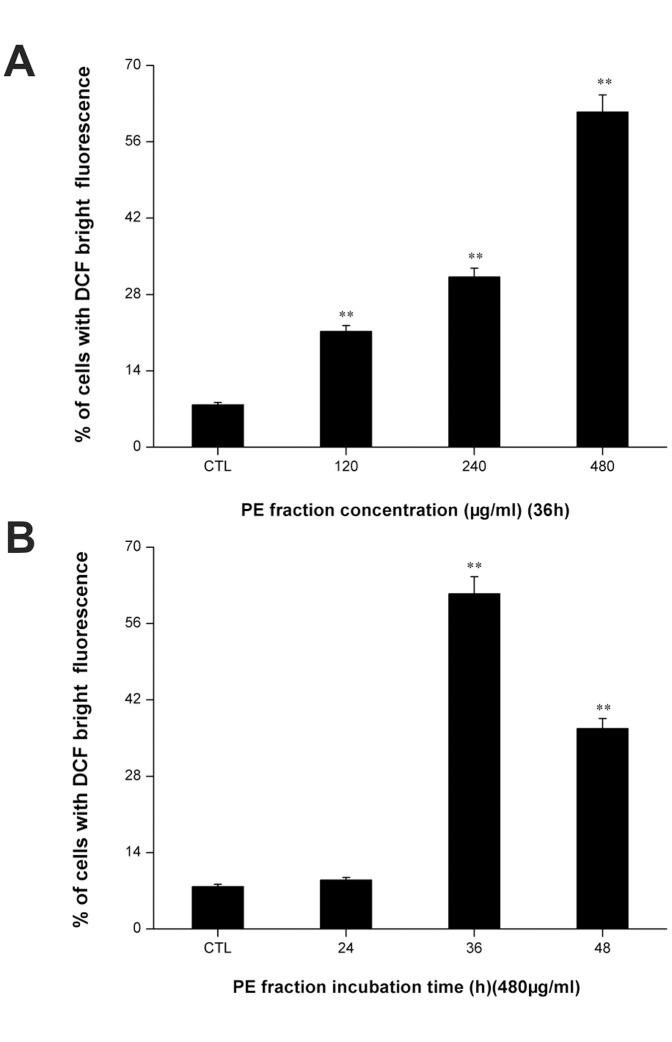
*Pyropolyporus fomentarius* PE fraction stimulated intracellular ROS generation in S180 cells. Cells were treated with different concentrations of PE fraction for 36 h (A), and treated at 480 µg/ml dose for various time periods (B), and labeled with DCFH–DA. The fluorescence intensity of the oxidized product DCF in individual cells was detected by flow cytometry. Each value is expressed as a mean ±SD of three independent experiments.**p<0.01 versus the control (CTL).

Because the generation of intracellular ROS may be related to the induction of apoptosis in various cell types, we explored whether the PFPE could stimulate ROS generation in S180 cells. Figure 6A shows that compared with controls, in a dose dependent manner, the generation of ROS dramatically increased in PFPE-treated cells (p<0.01), and, in the 480 µg/ml PFPE fraction treated group, at a 36 h incubation time, the intracellular ROS level was the highest (Figure 6B). These results demonstrated that PFPE markedly increased the intracellular ROS level.

### In vivo anti-tumor test

**Figure 7:**
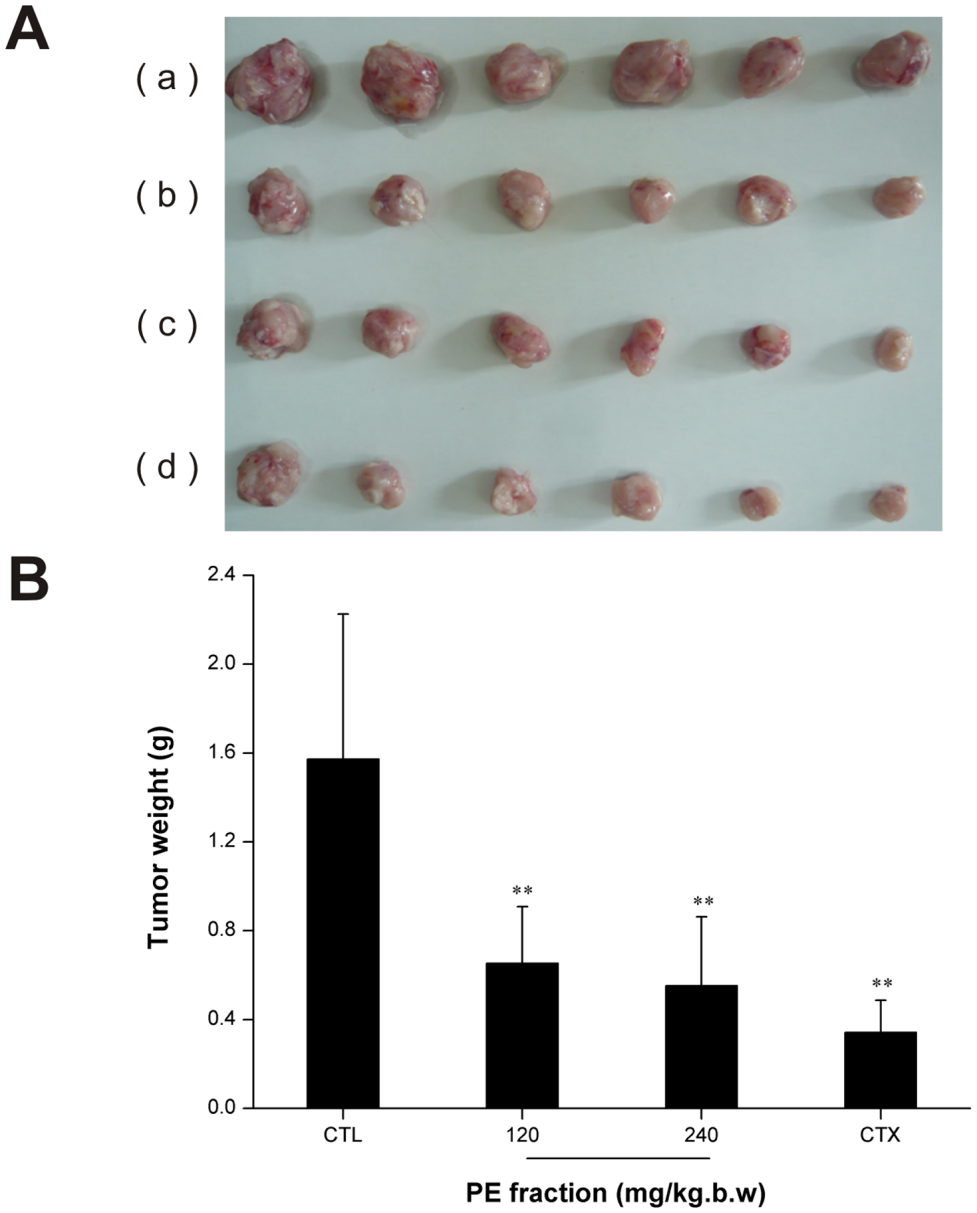
Tumors excised three weeks after treatment with *Pyropolyporus fomentarius* PE fraction from each mice group. (A) Image of tumors: (a) Control (CTL); (b) 120 mg/kg.d.w; (c) 240 mg/kg.d.w; (d) Cyclophosphamide (CTX), 20 mg/kg.d.w. (B) Determination of tumor weight, significant difference between control and treatment group was indicated by**p<0.01, values are mean±SD, n = 6.

Furthermore, our in vivo studies showed that the PFPE caused a significant decline in the sarcoma weight compared with the model control group (p<0.01) (Figure 7). The tumor inhibitory rate of the PFPE was 58.36% and 64.83% at the doses of 120 and 240 mg/kg, respectively. The spleen and thymus indexed are shown in Table 1.

**Table 1:** Organ index (mg/g BW) of repeated dose 3-weeks PFPE-treated mice^a^.

Group	Dose (mg/kg.d)	Spleen index	Thymus index
Model control	—	7.78±1.2	1.84±0.45
Low dose group	120	7.05±1.0	1.60±0.15
High dose group	240	7.05±0.97	1.27±0.33*
CTX	20	5.31±1.13*	1.29±0.32*

* p<0.05 vs model control group.

a Data are expressed as means ± SD (n = 6).

### Chemical compounds in PFPE

**Figure 8:**
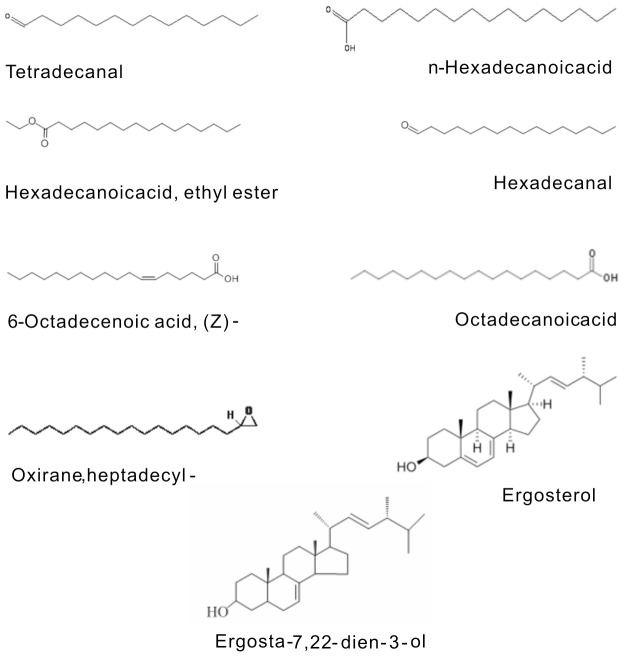
The structures of nine identified compounds in *Pyropolyporus fomentarius* PE fraction.

Upon GC–MS analysis, the chemical composition of the main constituents from PFPE was identified in Table 2. The main constituents were considered “identified” when their mass spectral fit values were at the default value of 90% or above. A total of nine constituents were identified in the fraction and the chemical structures of the identified compounds are shown in Figure 8. For the first time, our study reports the chemical compositions of the petroleum ether fraction from *P. fomentarius* fruit body by GC-MS analysis.

**Table 2:** Identification of *P*. *fomentarius* PE fraction metabolites using GC–MS analysis.

Retention time (min)	Compound	Peak area (%)	Molecular formula
17.457	Tetradecanal	1.34	C_14_H_28_O
19.599	n-Hexadecanoic acid	16.82	C_16_H_32_O_2_
20.025	Hexadecanoic acid, ethyl ester	1.09	C_18_H_36_O_2_
20.483	Hexadecanal	5.35	C_16_H_32_O
22.558	6-Octadecenoic acid, (Z)-	21.36	C_18_H_34_O_2_
22.967	Octadecanoic acid	17.11	C_18_H_36_O_2_
23.917	Oxirane, heptadecyl-	5.17	C_19_H_38_O
42.296	Ergosterol	4.17	C_28_H_44_O
42.546	Ergosta-7,22-dien-3-ol	3.46	C_28_H_46_O

## Discussion and Conclusion

The present work has demonstrated the significant anti-tumor activity of the PFPE both in vitro and in vivo.

One of the anti-tumor activity chemotherapeutic targets is cytotoxicity [35]. Most clinically used anti-tumor agents possess significant cytotoxic activity in cell culture systems. In our paper, PFPE was toxic to S180 cells at 240 and 480 µg/ml in time-dose-dependent manners. However, at the concentration of 120 µg/ml, there was no proliferation inhibition effect with the prolonged incubation time. The dose response phenomenon and low dose stimulation have been previously reported for other drugs [36]–[38]. The lower concentration might have other functions, such as anti-inflammation, anti-virus, etc., and the underlying mechanisms require further exploration. Moreover, PFPE had no or little cytotoxicity in HEK-293 cells. Thus, the present study suggests that PFPE has a potential application as a natural anti-tumor agent.

Defects in apoptosis are the critical step in the resistance to therapy in many types of cancers [39], [40]. Thus, apoptotic pathways are relevant targets in cancer therapies [41]. Previous studies have shown that apoptosis is an important mechanism through which various anticancer agents exert anticancer effects [42], [43]. In the present study, we aimed to determine whether apoptosis was induced in S180 cells along with the PFPE exposure. As evidenced by Annexin V-FITC/PI double staining, we found that the proportion of early and late apoptotic cells increased significantly after the PFPE treatment (Figure 2). Staining with DAPI then clearly showed that the PFPE caused fragmented punctate blue nuclear fluorescence and condensed chromatin in S180 cells (Figure 3), which are the morphological characteristics of apoptosis [44], [45]. As shown in Figure 4, PFPE caused obvious DNA fragmentation in S180 cells, which is also a typical biochemical feature of apoptosis [33]. The data in this study suggest that the PFPE could induce apoptosis in S180 cells, and the cytotoxic effects were associated with apoptosis, implying that the extract has great potential in anti-cancer drug screening.

Mitochondria play a critical role in cell apoptosis triggered by many stimuli [46], [47]. Loss of MMP is an early event in apoptosis. The MMP, as detected by flow cytometry with Rh123, significantly decreased immediately after 24 h PFPE treatment (Figure 5). Electron leakage from the mitochondrial respiratory chain may react with molecular oxygen, resulting in the formation of superoxide, which is subsequently converted to ROS [48]. Moreover, excessive ROS may cause oxidative damage to lipids, proteins, and DNA, leading to cell death. As seen from Figure 6, compared with the control group, the generation of intracellular ROS dramatically increased in S180 cells. These results suggest that the decreased MMP and elevated ROS may both related to cell apoptosis after the PFPE treatment, and the PFPE-induced cell apoptosis might be mitochondria dependent.

Consistent with in vitro findings, the in vivo study provides information that the PFPE significantly reduced the S180 sarcoma weight at the indicated dose (Figure 7), showing its specific role in anticancer therapy. In tumor immunotherapy, the occurrence, growth of tumor and immune state has a very important relationship [49]. Spleen and thymus are important immunological organs. The spleen index and thymus index could reflect the immune function of spleen and thymus. According to other reports [36], CTX caused atrophy of spleen and thymus and influenced the spleen and thymus indexes. We got similar results in the in vivo experiment in CTX treated group. However, at low concentration group, PFPE did not induce such changes (Table 1). As illustrated in our work, PFPE could efficiently inhibit tumor growth and also has lower immune organ toxicity.

Via GC–MS analysis, various possible chemical components were detected in PFPE (Figure 8). n-Hexadecanoic acid has anti-tumor activity to human leukemic cells as well as murine cells [50]. Hexadecanoic acid (palmitic acid) has been reported to induce NF-κB activation in HaCaT keratinocytes [51], which is an important pathway involved in cancer development. Moreover, fatty acids exhibit cytotoxicity against HeLa cells and retard tumor growth [52]. The essential oil, with n-Hexadecanoic acid and octadecanoic acid as the main components, showed significant cytotoxicity against oral cancer (KB), breast cancer (MCF-7) and small cell lung cancer (NCI-H187) [53]. Steroidal compounds had a potent inhibition effect against various cancer cells [54]–[57]. Therefore, the anti-tumor effect of PFPE both in vitro and vivo may be closely related to the specific efficacy of such components, functioning either alone or together, which needs further confirmation.

In conclusion, this work provided evidence that the PFPE inhibits the proliferation of S180 cells in vitro through inducing apoptosis, while the PFPE significantly reduced the weight of S180 sarcoma in vivo. Apoptosis induction has become a new therapeutic target in cancer research, and the results in our paper indicate that the PFPE may have a potential application as a natural anti-tumor agent. Future research should investigate the specific bioactive compounds and then try to explore the molecular mechanisms of the PFPE in cancer therapy.
